# Quality of life of caregivers of patients diagnosed with severe mental illness at the national referral hospitals in Uganda

**DOI:** 10.1186/s12888-016-1084-2

**Published:** 2016-11-15

**Authors:** Cynthia Ndikuno, Mariam Namutebi, Job Kuteesa, David Mukunya, Connie Olwit

**Affiliations:** 1 Department of Nursing, College of Health Sciences, Makerere University, Kampala, Uganda; 2 Department of Surgery, College of Health sciences, Makerere University, Kampala, Uganda; 3 Department of Pediatrics, College of Health Sciences, Makerere University, Kampala, Uganda; 4 Department of Nursing, College of Health Sciences, Makarere University, Mulago Hill road, P.O BOX 7072, Kampala, Uganda

**Keywords:** Caregivers, Quality of life, Severe mental illness, WHOQOL-BREF, Uganda

## Abstract

**Background:**

Worldwide, 450 million people suffer from mental and behavioral disorders. In Uganda, it is estimated that 35% of the population that is 9,574,915 people suffer from some form of mental illness. Caregivers are increasingly bearing the responsibility of taking care of these patients, which can influence their QoL due to the social and economic costs they incur. The aim of the study was to assess the QoL of caregivers for patients diagnosed with severe mental illness attending the National Referral Hospitals in Uganda.

**Method:**

This was a cross sectional study. A pretested tool with two parts; a sociodemographic part and a validated WHOQOL-BREF, was used to collect data from 300 consecutive eligible participants. SPSS (Statistical Package for Social Sciences) Version 22 and Stata Version 14 were used in data entry and analysis.

**Results:**

Of the 300 participants, 57.3% of the caregivers had a poor QoL. The statistically significant factors associated with QoL were environment (Adjusted coefficient = 0.016, 95% CI = 0.009–0.023), caregiver satisfaction with their health (Adjusted coefficient = 0.405, 95% CI = 0.33–0.487), psychological wellbeing (Adjusted coefficient = 0.007, 95% CI = 0.0002–0.013), and education level (Adjusted coefficient = 0.148, 95% CI = 0.072–0.225).

**Conclusion:**

QoL of caregivers for patients diagnosed with mental illness is generally poor due to the added responsibilities and occupation of their time, energy and attention. This additional responsibility results in high levels of stress and caregivers may fail to have appropriate coping mechanisms. Interventions like support groups or counseling should be put in place to aid caregivers in their role and therefore improve QoL. This study adds to the international database of QoL literature and calls for more attention to be placed on caregivers in supporting their role and improving their QoL so as to lead to better patient outcomes among those diagnosed with mental illness.

## Background

Globally, mental health disorders are known to represent two of the ten leading causes of disability and account for 35.6% of the total burden of disease according to the Global Burden of Disease (GBDCollaborators) Collaborator’s study [1]. In Uganda, it is estimated that 35% of the population that is 9,574,915 people suffer from some form of mental illness with atleast 15% requiring treatment [2].

In an African perspective, the individuals diagnosed with mental illness are cared for in the community by caregivers who may be relatives or friends [3]. Caregivers offer care that may be physical, economical, psychological, and social which eventually changes their life since it is involving and demanding [4].

Caregivers may experience reduced productivity at home and in the workplace thus losing wages [5]. This combined with the health care costs for the patients diagnosed with severe mental illness affect the caregivers’ financial situation thus creating or worsening poverty [5]. Caregivers are also prone to experiencing social consequences including; disrupted social networks, stigma and discrimination, which exposes them to high levels of depression, stress and anxiety [5, 6]. These social, economic and psychological changes may greatly impact on the caregivers’ Quality of Life (QoL) [7].

QoL is an individual’s perception of their position in life in the context of the culture and value systems in which they live and in relation to their goals, expectations, standards and concerns.

The QoL of caregivers is important in reflecting the quality of care that is given to patients diagnosed with mental illness to aid recovery or stability and can also affect the progress and outcome of the patients [6].

Caregivers are fundamental in giving care to patients diagnosed with severe mental illness however there is limited documentation on the QoL of the caregivers for these patients. Due to the limited documentation of QoL, there are few interventions for caregivers that are incorporated in the Uganda health policy to help them handle with their role [8]. The few interventions include the community mental health program run at Butabika Hospital that offers caregiver counseling to people living within the 10 km radius of the hospital and the Schizophrenia fellowship caregiver support group found in Jinja district. Therefore it is important to understand their QoL, which may give baseline information on their way of life and guide health workers and policy makers on the development of appropriate interventions to support the caregivers in their role.

This study sought to determine the QoL and associated factors of caregivers of mentally ill patients attending the National Referral Hospitals in Uganda.

## Methods

### Study site

This study was conducted at the National Referral Hospitals of Uganda including Mulago Hospital and Butabika Hospital. These national referral hospitals are located in Kampala district in Uganda and they are both teaching hospitals.

Mulago hospital has an Outpatient mental health clinic known as S.B Bosa Mental Health Unit. On the other hand Butabika Hospital is a psychiatric hospital with inpatient wards and an outpatient clinic. The patients usually come with their caregivers.

### Study design

This was a descriptive cross sectional study of caregivers for patients diagnosed with mental illness.

### Study population

The study population comprised caregivers of patients diagnosed with severe mental illness including Schizophrenia, Bipolar Affective Disorder and Major Depressive Disorder who were attending S.B Bosa Mental health outpatient clinic in Mulago Hospital, the Outpatient clinic in Butabika hospital and the caregivers who were visiting the people they care for that were admitted on the inpatient wards in Butabika Hospital.

### Sample size

As recommended by the WHOQOL-BREF (World Health Organisation Quality of Life-BREF) manual, the sample size used in this study was 300 caregivers [9].

This is considered an adequate sample size by the WHOQOL Group to give significant results that can be used in establishing the relationships between the independent and dependent variables.

### Sampling procedure

All eligible caregivers who consented to participate were enrolled consecutively in the study from January 2015 to May 2015 until the required sample was obtained.

The participants included in the study were caregivers for a patient diagnosed with severe mental illness including Schizophrenia, Bipolar Affective disorder and Major Depressive disorder; caregivers who had taken care of a patient diagnosed with mental illness for atleast six months; caregivers who consented to participate in the study and caregivers who were above 18 years of age.

The caregivers who were excluded had people diagnosed with severe mental illness who were aggressive or had the potential to harm themselves or people around them and those who were unable to stay around the clinic long enough to fill in the tool.

### Data collection tools

A pre-tested data collection tool was used to collect the data from the caregivers. The tool had two parts with the first part seeking Sociodemographic and the second part being the WHOQOL-BREF.

The sociodemographic section had questions on age, sex tribe, religion, occupation, monthly income, education level, marital status, period of time spent caring for the patient diagnosed with mental illness and the mental illness the patient is diagnosed with.

The WHOQOL-BREF is a 26 item, self-administered questionnaire (it can also be interviewer assisted or interviewer administered), which contains a total of 24 questions and two items that measure overall QoL and general health. Participants express how much they have experienced the items in the preceding 2 weeks on a 5-point Likert scale ranging from 1 (not at all) to 5 (completely) and the administration time is usually 10–15 min.

The WHOQOL-BREF contains 2 items from the overall QoL and general health facet and 1 item from each of the remaining 24 facets making a total of 26 question items. The 24 facets of QoL make up the four domain s namely: physical health that comprises 7 items, psychological wellbeing that comprises 6 items, social relationship that comprises 3 items, and environment that comprises 8 items. Question 1 and 2 plus the domain scores were calculated and transformed using WHOQOL-BREF SPSS syntax file. The transformed scores were on a scale of 0–100. In this study, all items that were rated with a higher score indicated a higher or better QoL [9]. The internal consistency of the various multi-item domains used in this study was assessed in an international field trial involving 23 countries representing various regions and cultures [10]. The Internal consistency was found to high with an average Cronbach’s alpha of 0.81 for the physical health, psychological wellbeing and environmental domains but a marginal score of 0.68 for social relationship domain.

The WHOQOL-BREF was translated to Luganda and field tested among a sample of people living with or without HIV in Uganda [11] as part of the validation process. The results showed that the Luganda version of the WHOQOL-BREF had acceptable validity and reliability of 0.915.

### Study procedure

Approvals were obtained to collect data in Mulago Hospital SB Bosa Mental health clinic and Butabika hospital from the heads of department and the nurses in charge of the wards. All participants were screened for eligibility and eligible caregivers were recruited consecutively until the desired sample size was obtained. The eligible caregivers were taken to a room within the facility to maintain privacy, the study was explained to them and informed consent obtained from them. This was done before the patients went to see the psychiatrist for review and treatment or before the caregivers commenced their visits with the patients who were admitted on the wards. This is because of the long time which the caregivers spent before they were seen by the psychiatrist.

Data was collected from the caregivers and their patients’ medical records using the interviewer-administered tool. The time required for the interview was approximately 15–20 min. For those who did not understand English, a Luganda version of the tool was used. There was no immediate benefit or compensation for the caregivers’ time.

### Data analysis plan

Data was analyzed using SPSS Version 22 statistical software and Stata. The total QoL score was calculated and transformed using SPSS syntax file and the final scores were categorized as good QoL and Poor QoL. Caregivers with scores above 50 were categorized as having good QoL.

Univariate analysis was performed for caregiver factors of the study participants, which were categorized and presented as categorical variables. For categorical variables proportions and percentages were reported and findings displayed in frequency distribution tables.

For continuous variables such as age, mean and standard deviations were reported. The WHOQOL-BREF domain scores were reported in a table reflecting minimum and maximum scores, the mode, mean and median. The QoL was presented in a pie chart with the percentages for poor and good QoL.

Bivariate and multivariate analysis was performed to assess the relationship of associated factors with QoL using the linear regression method to derive the coefficients with associated confidence intervals. The associated factors were in domains that were scored and the sociodemographic characteristics. The scalar or continuous variables were used in bivariate analysis and these include; age, physical health, psychological wellbeing, social relationship and environment. Associations or relationships from bivariate analysis with *p* values less than 0.2 and variables that were associated with QoL from our literature search were considered for stepwise linear regression model using Stata. The stepwise regression was used to identify confounders that were variables that caused a difference of ≥ 10% between the unadjusted and adjusted coefficients. The confounders were dropped and variables that had a strong relationship with the outcome variable were maintained. The variables with *p* values less than 0.05 were considered statistically significant in association to QoL.

## Results

### Sociodemographic characteristics of the participants

A total of 300 participants were interviewed using interviewer-administered questionnaires. The response rate for this study was 0.94. 128 (42.7%) of the participants were male and 172 (57.3%) were female and the age ranged from 18 to 75 years. The mean age was 36.59 (SD ± 0.73) and 111 (37.0%) participants were within the age group of < 30 years. Majority of the participants, 163 (54.3%), were Baganda and the most common religion was Catholic that constituted 143 (41.3%) (See Table 5 in Appendix section).

The most common diagnosis of the patients was Bipolar Affective Disorder, which constituted 45%. Of the 300 caregivers; 123 (42.3%) had taken care of their relatives or friends diagnosed with mental illness for a period of time between 1 year to <5 years (see Table 6 in Appendix section).

A few caregivers had missing data for income, educational level and marital status. This is because they did not feel comfortable giving the information and considered it sensitive.

### Caregivers’ satisfaction with health

Concerning the caregivers’ satisfaction with health, 146 (48.67%) were very dissatisfied and 154 (51.33%) were satisfied with the state of their health (see Table 1 below).

**Table 1:** Caregiver satisfaction with their health

Level of satisfaction	Frequency	Percentage (%)
Very dissatisfied	24	8.0
Dissatisfied	59	19.7
Neither satisfied nor dissatisfied	63	21.0
Satisfied	137	45.7
Very satisfied	17	5.7

### WHOQOL domain scores

There are four WHOQOL domains that include physical health, psychological wellbeing, social relationships and environment. Each of these domains was scored and the mean scores for each domain were; physical health 52.20 (SD ± 15.90), psychological wellbeing 55.97 (SD ± 15.94), social relationships 51.64 (SD ± 21.08) and environment 50.9 (SD ± 17.35) (see Table 2 below).

**Table 2:** The Caregivers’ WHOQOL domain scores

Domain	Minimum score	Maximum score	Mode	Median	Mean	SD
Physical health	10.71	100	64.29	57.14	55.20	15.90
Psychological	12.50	91.67	50.00	58.33	55.97	15.94
Social relationships	0.00	100	58.33	50.00	51.64	21.08
Environment	0.00	96.88	56.25	53.13	50.49	17.35

### Quality of life of caregivers

The QoL was measured on a scale of 1–5 from very poor to very good where 1 indicated very poor and 5 indicated very good. The scores 1–5 were then transformed using the WHOQOL-BREF syntax file to a scale of 0–100. The QoL measure was further categorized based on the score onto two parameters: good and poor QoL. Scores > 50 represented a good QoL while scores ≤ 50 represented a poor QoL. Of the 300 caregivers, 172 (57.30%) had a poor QoL while 18 (42.70%) had good QoL.

### Factors associated with the QoL of caregivers of patients diagnosed with mental illness

Bivariate analysis and multivariate analysis of factors associated with QoL was done using linear regression to determine the factors significantly affecting QoL. The variables in this study fulfilled the assumptions for linear regression since they had a linear relationship, and multivariate normality. The variables also had no auto-correlation and no multicolinearity between them the highest VIF being 2.191.

According to Table 3 below, the QoL decreased with increasing age of the participants (Unadjusted Coefficient = −0.018, 95% CI = −0.027 – -0.009). QoL increased with increasing scores of the WHOQOL-BREF domains including physical health (Unadjusted Coefficient = 0.033, 95% CI = 0.027 – 0.039), psychological wellbeing (Unadjusted Coefficient = 0.036, 95% CI = 0.030 – 0.042), environment (Unadjusted Coefficient = 0.038, 95% CI = 0.033 – 0.043), and social relationships (Unadjusted Coefficient = 0.74, 95% CI = 0.527 – 0.957). All these factors were statistically significant in association with QoL with *p* values < 0.001.

**Table 3:** Bivariate analysis of individual factors associated with QoL of caregivers

Variable	Unadjusted coefficient	95% CI	*p* value
Age	−0.018	−0.027 – -0.009	<0.001*
Physical health	0.033	0.027 – 0.039	<0.001*
Psychological wellbeing	0.036	0.030 – 0.042	<0.001*
Environment	0.038	0.033 – 0.043	<0.001*
Social relationship	0.74	0.527 – 0.957	<0.001*

*Statistically significant since *p* < 0.05

However the above factors plus the categorical variables including gender, monthly income, period of time spent caring for the patient, education level, marital status and caregiver satisfaction with health were adjusted for in multivariate stepwise linear regression model (Table 4 below). Out of the 11 independent variables entered in the model, 7 variables were dropped due to their confounding effect. The confounders included; age, gender, monthly income, period of time spent caring for the patient, marital status, and physical health. The statistically significant factors associated with QoL that were derived included; environment (Adjusted coefficient = 0.016, 95% CI = 0.009 – 0.023), caregiver satisfaction with their health (Adjusted coefficient = 0.405, 95% CI = 0.33 – 0.487), psychological wellbeing (Adjusted coefficient = 0.007, 95% CI = 0.0002 – 0.013), and education level (Adjusted coefficient = 0.148, 95% CI = 0.072 – 0.225).

**Table 4:** Multivariate analysis of factors associated with poor QoL of caregivers of patients diagnosed with mental illness

Variable	Adjusted coefficient	95% CI	*p* value
Environment	0.016	0.009 – 0.023	<0.001*
Caregiver satisfaction with their health	0.405	0.33 – 0.487	<0.001*
Psychological wellbeing	0.0065	0.0002 – 0.013	0.044*
Education level	0.148	0.072 – 0.225	<0.001*
Social relationship	0.003	−0.001 – 0.007	0.180
Constant	0.231	−0.084 – 0.547	0.150

*Statistically significant since *p* < 0.05

The final model had n R^2^ (variance) of 0.6 and an F static value of 85.6 with *p* < 0.00. This showed that the linear regression model used had adequate explanatory power for the relationships observed between the independent and dependent variables in this study.

## Discussion

### QoL of caregivers of patients diagnosed with mental illness

In this study we found that of the 300 participants, 172 (57.30%) had a poor QoL. These results are consistent with the different studies done in Hong Kong and Europe that suggested the QoL of caregivers is poor in comparison with the general population [7, 12]. QoL research is rare in sub-Saharan Africa [13], which makes it difficult to find comparable studies among the African population.

This poor QoL of life is probably due to the task of caregiving, which results in additional responsibilities on the caregivers’ daily life, and occupies their time, energy, and attention. This additional responsibility results in high levels of stress that impact negatively on the QoL of the caregivers [14, 15]. Due to this additional responsibility and high levels of stress, caregivers may fail to have appropriate means of dealing with this added role [16]. This puts the caregivers at risk of developing psychosocial or physical illness therefore interventions should be put in place to aid them in dealing with their role and thus improve their QoL [14].

### Factors associated with QoL of caregivers of patients diagnosed with severe mental illness

Caregiver satisfaction with health, education level, psychological wellbeing and environment were the four main factors, which were significantly associated with QoL of caregivers of patients diagnosed with severe mental illness.

The caregivers who were dissatisfied with their health were more likely to have a poor QoL. This is consistent with the results of a study done in China which reflected that caregivers sacrifice themselves to care for their patients, resulting in strain on their physical and mental health [17]. This dissatisfaction in health is influenced by the inadequate time the caregiver spends on their health concerns since most of their time is invested in their role. As caregivers adjust their social lives to the needs of patients diagnosed with mental illness, they usually have concerns about their own health but neglect them therefore their health deteriorates which eventually impacts on their perception of their overall QoL [18].

It was suggested that people with good health status may have a better QoL [19] since they are usually satisfied with the state of their health. Those with a low health status are usually dissatisfied with their health since they have anxiety about their own health and the health of their patients.

Professional health care providers can have a significant impact on the health and well-being of caregivers [6]. Therefore health workers should be vigilant in identifying healthcare problems early in this group so as to manage them adequately. This can be done when the caregivers visit the hospital with their patient or while they are on the inpatient ward. Health care programs can be put in place to raise awareness of the important role of caregivers in management of patients diagnosed with mental illness among health care providers so that they are not ignored during the routine clinic or hospital visits.

The education level of the caregiver was positively correlated with QoL showing that the higher the education level attained by the caregiver, the better their chances of having a good QoL.

These findings are consistent with the findings of studies carried out in Hong Kong and Turkey [6, 7]. As caregivers attain higher levels of education, they become more knowledgeable and develop more effective skills in managing the demand that comes with their role thus improving their QoL. For example they can be able to read and understand basic instructions required to take care of patients diagnosed with severe mental illness, which makes their role easier. Highly educated caregivers tend to have better paying jobs or sources of income and are able to adequately use their financial and social resources available in their communities to deal with the caregiving burden resulting in better QoL.

The Ugandan education system is comprised of three major levels including primary, secondary and tertiary [20]. The primary level includes students from 6 to14 years, secondary includes 14 to 19 years and tertiary level includes students from 19 to 24 years. Since basic education is a fundamental human right and a component of wellbeing, the government of Uganda has put in place certain measures to avail education to all citizens. This has been done through introduction of Universal Primary Education (UPE) and Universal Secondary Education. The schools under these programs offer free education for those who may not be able to afford paying for it. This free education leads to increase in number of literate caregivers therefore leading to improvement in QoL.

Concerning psychological wellbeing, the results showed that the lower the score, the lower the QoL of the caregiver. Low scores reflected a poor psychological wellbeing. The caregiving role usually adds responsibilities on the caregivers’ daily life, and occupies their time, energy, and attention [21]. This results in caregiver stress and strain and predisposes them to development of mental or psychological illness especially if they have genetic predisposition [14, 16].

The most common psychological illnesses observed among caregivers include depression and anxiety disorders [6, 22]. Inorder to improve their psychological wellbeing and avoid development of any mental disorders, caregivers require social support or assistance in caring for their relatives or friends that are diagnosed with severe mental illness. This assistance ensures that caregivers are left with enough time and resources to address their own psychological needs [19]. Psychological wellbeing can be improved through support groups where caregivers are able to share the stressors related to their role so that they can adequately handle their role [8]. Support groups usually offer instrumental and emotional support. Improved psychological wellbeing results in better QoL of caregivers and they are able to carry out their role so that the patients diagnosed with mental illness can have good health outcomes.

Environment domain score had a positive correlation with QoL of caregivers. The environmental domain was assessed using a number of components which included: financial resources, freedom, physical safety and security, health and social care, home environment, opportunities for acquiring new information and skills, participation in recreation/leisure activities, physical environment and transport. These components may not significantly influence the QoL on their own but have an effect in combination. The significance of the environment’s influence on QoL of caregivers is consistent with a study done in China [19].

In Uganda, certain sociocultural aspects dictate the caregiving role. The responsibilities of a caregiver are carried out both in the hospital setting and in the community [8].

While in the hospital after admission of the patient diagnosed with mental illness, the caregiver ensures that the patient’s basic needs are met from feeding to washing clothes, bathing and hygiene. Doctors and nurses utilise the caregivers in administering medication and monitoring the patients. Occasionally when drugs are unavailable in the hospital, the caregiver is incharge of purchasing them from pharmacies or drug shops within the community. While in the community after discharge, caregivers ensure physical wellbeing of the patient by providing the basic needs, protecting them from harm and watching them so that they do not wander and get lost or go missing. Caregivers also ensure that the patients adhere to drugs and go for their follow-up appointments or visits in the mental health clinics. Caregivers also offer emotional and social support to the patients diagnosed with mental illness.

Opportunities for acquiring new information concerning the illness of the patient and how to care for the patient in day to day life is important and it reduces the caregivers sense of strain or burden in their role [19]. As ones financial resources increase, their ability to afford care and hospitalization plus medication for their patients increases while strain and stress in caring role is reduced. Drug banks have been created in caregiver support groups where money is pooled and drugs are bought in bulk cheaply thus aiding the caregiver role. Therefore caregivers require information, support, good communication with mental health services and adequate financial resources inorder to effectively handle caring for the patients diagnosed with mental illness [12].

### Limitations

This study had a number of limitations including cultural bias as some participants feared to answer questions about their sex life and intimate or personal relationships. The results of this study cannot be generalized to the entire population of caregivers worldwide due to differences in culture and environment in different areas.

## Conclusion

The QoL of the majority of caregivers of patients diagnosed with mental illness attending the National Referral Hospitals in Uganda is poor. From this study the main factors associated with the QoL are caregiver satisfaction with health, environment, education level and psychological wellbeing. Attention to these factors should therefore be considered central to any intervention for caregivers in this population.

Nurses and other health care professionals therefore need to be aware of the poor QoL of caregivers so as to put in place interventions like support groups or passing on information about the mental illness and how to care for the patients diagnosed with mental illness in order to contribute to improving the QoL of caregivers.

This research done in a low-income country setting adds to the body of evidence that has accrued from high-income country settings to call for increased attention to caregivers for patients diagnosed with mental illness. Internationally, interventions need to be put in place to aid caregivers in their role based on the sociocultural setting in the different countries.

### Implications for practice

Health workers should be aware of the poor QoL among caregivers so as to routinely measure this QoL and intervene where there is impairment. Support groups should also be put in place for the caregivers where they can share and assist each other to reduce the burden of care thus improving their QoL. Within these support groups they can so being drug banks or collection of funds that can be used to aid caregivers that may be lacking resources to use in their role. In addition, counseling sessions should be put in place for the caregivers at the different health facilities or within the community.

The policy makers at different government levels should put in place policies that ensure integration of caregiver interventions within the mental health system. Community mental health programs should also be integrated in the different decentralized health facilities where awareness about mental illness can be increased among the community members and thus reduce stigma against patients and their caregivers. The increased awareness can so lead to provision of social support to caregivers by the community members thus reducing social isolation among the caregivers and eventually leading to better QoL.

Further research should be carried out to assess the relationship between caregiving situation (objective and subjective burden) and QoL of caregivers. Further research should also be carried out involving a case control study to compare QoL of caregivers of patients diagnosed with severe mental illness with the QoL of the general population.

## Appendix

**Table 5:** Socio-demographic characteristics of the study participants

Variable	Frequency (*N* = 300)	Percentage (%)
Age		
≤ 30	122	40.7
31–40	77	25.7
41–50	56	18.7
51–60	24	8.0
≥ 60	21	7.0
Gender		
Male	128	42.7
Female	172	57.3
Tribe		
Baganda	163	54.3
Banyankole	20	6.7
Basoga	25	8.8
Others^b^	92	30.7
Religion		
Catholic	124	41.3
Moslem	42	14.0
Pentecostal	49	16.3
Protestant	71	23.7
Others^c^	14	4.7
Marital status (*N* = 290)^a^		
Single	100	34.5
Married	111	38.3
Living as married	44	15.2
Separated	15	5.2
Divorced	12	4.1
Widowed	8	2.8
Occupation		
Housewife	37	12.3
Subsistence Farmer	27	9.0
Self-employed	95	31.7
Professional	51	17.0
Unemployed	63	21.0
Others^d^	27	9.0

^a^10 participants did not answer this question

Others ^b^stands for other tribes including Itesot, Banyoro, Madi, Dinka, Batoro, Bagisu, Lugbara, Banyala, Japhadola, Alur, Acholi, Langi, Banyarwanda, Bakonjo, Bafumbira, Bakiga and Basamya. Others ^c^for other religions including SDA, Orthodox, Moon-Worshipper and Baptist. Others ^d^stands for other occupations including student, construction workers and pastor

**Table 6:** Other Sociodemogragraphic characteristics of caregivers

Variable	Frequency (*N* = 300)	Percentage
Education level (*n* = 288)^a^		
None at all	29	10.1
Primary	61	21.2
Secondary	95	33.0
Tertiary	103	35.8
Income (*n* = 291)^b^		
Shs. 0–50,000	154	54.2
Shs. 50,001–100,000	41	14.4
Shs. 100,001–500,000	63	22.2
Shs. 500,001 and above	26	9.2
Diagnosis of the patient		
Schizophrenia	105	35
Bipolar Affective Disorder	135	45
Major Depressive Disorder	60	20
Period of time spent in taking care of the patient		
< 1 year	63	21.6
1 year to < 5 years	123	42.3
5 years to < 10 years	49	16.8
10 years to < 20 years	41	14.1
20 years and above	15	5.0

^a^12 participants did not answer the question. ^b^9 participants did not answer this question
